# Formal Education and Migration Aspirations in Ethiopia

**DOI:** 10.1111/padr.12159

**Published:** 2018-07-01

**Authors:** Kerilyn Schewel, Sonja Fransen

Expanding formal education is a universal and uncontested aim of development policy. The last several decades have ushered in dramatic shifts in access to schooling, particularly at the primary and secondary level. Gross enrollment ratios in primary school in low‐income countries, for example, grew from 46 percent in 1970 to over 100 percent in 2015 (World Bank 2017).^1^ Alongside its intrinsic value, the social benefits of widening access to formal education are well known: education tends to expand economic opportunity, promote health, and contribute to greater gender equality (Benavot 2006). However, the impact of rising access to formal education on migration has received comparatively little attention. Migration, unlike income, health, or gender equality, is often not perceived as a universal good. On the contrary, increased political interest in migration focuses on reducing the internal and international mobility of the world's poor. Governments and non‐governmental institutions alike, across a wide variety of contexts, perceive growing rural‐urban migration as a challenge to rural futures and urban sustainability, the international emigration of skilled workers as a loss for national development (the so‐called “brain drain”), and the emigration of unskilled workers as a threat to security and national identity in destination countries. This paper considers the mobility consequences of expanding formal education in Ethiopia. In particular, it examines the impact of primary and secondary education on the migration aspirations of young people.

Disentangling the links between migration, education, and aspirations is important for Ethiopia and other countries in sub‐Saharan Africa that place agriculture at the center of strategies for inclusive economic growth and poverty reduction. Excitement about the future of agriculture in Africa exists alongside a growing concern for whether young people desire to be a part of it (Leavy and Smith 2010; Tadele and Gella 2014). Africa's agrarian workforce is aging, and more and more young people aspire to urban, non‐agrarian livelihoods. Although opportunities in industry and services are increasing across the continent, the capacity for these sectors to employ significant shares of the population remains limited. There may be a mismatch between development strategies that focus on agricultural development and the actual livelihoods young people wish to pursue (Anyidoho et al. 2012).

The most common explanations for the reluctance of young generations to pursue agricultural livelihoods are structural and include: population growth and land scarcity; lack of government investment in small‐scale agriculture or rural infrastructure; economic shifts that make small‐scale agricultural less and less viable, even if more productive than for previous generations; insufficient rural job creation; and environmental degradation (White 2012; Leavy and Smith 2010; Sumberg et al. 2014; Tadele and Gella 2012; Bezu and Holden 2014; Mohammed 2016). These explanations, contested as they may be, paint the shift away from agriculture as resulting from negative forces that “push” young people out of agrarian lives.

We argue that alongside these structural explanations is an underappreciated change in young people's aspirations and expectations for the future, influenced by what is generally regarded as a positive development: the rise of formal schooling. Sociologists have long argued that modern education is an “agency of socialization” through which, in addition to learning knowledge and skills, children are taught particular norms and attitudes toward work and society (Durkheim 1956; Parsons 1964). White argues that formal schooling, as it is currently practiced in rural areas, “teaches young people not to want to be farmers” (White 2012: 12). Others suggest formal education tends to inflate aspirations and expectations for the future, creating a gap between young people's professional aspirations and the opportunities that are available to them in rural areas (Sumberg et al. 2014). The International Fund for Agricultural Development (IFAD) has called for a new type of curriculum, arguing that “agriculture must be accorded prestige, and sustainable agricultural intensification must be recognized and presented as modern and profitable, so that the aspirations of rural youth—girls as well as boys—can converge around it” (IFAD 2011: 171). Changing aspirations, shaped by schooling, appear to be a significant force shaping migration trajectories toward urban centers or abroad.

Ethiopia provides a particularly interesting case to study the influence of formal education on changing aspirations. The current generation of Ethiopian youth is the first to receive access to primary education on a wide scale; net enrollment rates increased from 21 to 93 percent over the last two decades (UN 2014). This rapid educational transformation seems to coincide with another important shift: an aspirational orientation away from rural, agrarian livelihoods toward urban, professional futures (see Abebe 2008; Tadele and Gella 2012; Camfield 2011; Maurus 2016). Although Ethiopia remains one of the least urbanized countries in Africa—with some 80 percent of its population living in rural areas, compared to an average of 62 percent in sub‐Saharan Africa—the country is urbanizing quickly, at a rate of 4.9 percent (2010–2015 estimate), and the economic, educational, and demographic transitions unfolding in Ethiopia augur significant shifts for the movement of its populations.

This paper proceeds in three parts. First, we examine how aspirations and education have been treated within migration theory to date and the explanations offered about why participation in formal education may increase the likelihood of migration. Second, we review the development context in Ethiopia and, using Labour Force Survey data, provide an overview of internal migration over the past two decades and the educational backgrounds of recent migrants. Third, we use survey data from the fourth round of the Young Lives study conducted in Ethiopia in 2013–2014 (Boyden et al. 2016) to map the internal and international migration horizons of rural and urban youth. We explore how educational attainment—alongside other potentially related development indicators, such as wealth, employment, and levels of self‐efficacy—relates to the aspiration to live elsewhere.

## Aspirations and education in migration theory

Research on aspirations has recently come in vogue in both the migration and development fields, largely because the concept extends our understanding of decision‐making beyond the limitations of rational choice assumptions. Rather than being the outcome of simple cost‐benefit analyses, aspirations are better understood as the subjective hopes and goals that guide decision‐making processes, setting the horizons within which life choices are made. Aspirations are not formed in isolation; they are fundamentally social, shaped by our experiences and observation of others within a cultural context (see Appadurai 2004; Bandura 1977; Sherwood 1989).

In the field of development, researchers study aspirations in relation to economic decision‐making, levels of self‐efficacy (i.e. one's confidence in one's ability to succeed; see Bandura 1997), and, using an old but increasingly relevant term today, “achievement motivation” (McClelland 1961; Ray 2006; Dalton, Ghosal and Mani 2015; World Bank 2015). Some of the most explicit development interventions aimed at shaping aspirations have been carried out in Ethiopia, where researchers suggest low aspirations and fatalistic beliefs are obstacles to development (Bernard et al 2011; 2014; Korten and Korten 1972; cf. Tafere 2014). In migration studies, researchers approach aspirations from two angles: people's broader life aspirations that directly or indirectly affect migration decision‐making; and migration aspirations per se, referring to the conviction that migration is preferable to staying (Carling 2014; De Haas 2014). Growing interest in the latter is signaled by the rise in surveys that seek to capture migration aspirations in origin countries (Gallup 2008; Van Dalen et al. 2005; Wood et al. 2010; Becerra 2012; Creighton 2013), though not without some conceptual difficulties (see Carling and Schewel 2018). One limitation to these surveys—something we overcome in our analyses here—is the almost exclusive focus on international migration aspirations, which misses the diversity of migration trajectories considered by potential migrants, within their country or without.

Before considering how education influences migration aspirations, it is worth reviewing how migration theories treat education more generally. Though patchy and somewhat scattered, migration theories generally hypothesize that rising educational attainment should increase the likelihood of migration. These theoretical explanations may be viewed from three related perspectives. The first set of perspectives focuses on migration decision‐making and describes education as a form of human capital that increases the potential gains available to an individual at a potential destination. When decision‐making is presented as a cost/benefit analysis—in which potential migrants weigh the economic costs and benefits of movement and then migrate where expected net returns are greater than estimated costs over a period of time—this perspective predicts that higher education levels should boost the expected benefits and thus likelihood of migration (see Sjaastad 1962; Schwartz 1976; Borjas 1990; Massey et al. 1993).

The second set of perspectives focuses on the structural dynamics of modern labor markets, which concentrate skilled labor in urban areas. The more specialized the skill set, the narrower the economic opportunity available and the greater the geographic spread of the labor market an individual must consider. As Lee (1966: 53) hypothesized: “The aim of prolonged education is to create specialists, for many of whom the demand is small in any one place but widespread. For them, migration is a concomitant of their vocations. Thus, engineers and professors have become peripatetic, but so have business executives and actors.” Nevertheless, some research finds important differences between internal and international labor markets. Higher educated individuals may be less likely to migrate internationally if their credentials do not transfer fluidly, due to differences in language, culture, and economic and educational systems (Taylor 1987).^2^ This suggests that while we may expect internal migration to present a positive linear relationship with education, international migration may have different dynamics.

A third perspective, overlapping with the previous two, presents expanding formal education as a potential driver of both the aspiration and capability to migrate. A growing number of studies, referred to as “two‐step approaches” to migration (Carling and Schewel 2018), separate the migration process along two dimensions: the evaluation of migration as a potential course of action and the realization of actual mobility or immobility at a given moment (e.g. Carling 2002; Docquier, Peri and Ruyssen 2014; Koikkalainen and Kyle 2015; Creighton 2013; Coulter et al. 2011). In this regard, De Haas (2007; 2014) proposes one hypothesis about the influence of education on each of these “steps” within the migration‐development nexus. Detailing an “aspiration‐capability approach,” he argues that in the short‐to‐medium term, processes of development—particularly growing access to formal education, information, and the media—tend to increase the aspiration to migrate, while greater infrastructure development, connectivity, and access to financial, social, and human capital increase potential migrants’ capabilities to leave, whether for internal or international destinations (De Haas 2007; 2014). According to De Haas (2014), aspirations are a function of people's general life aspirations and perceived spatial opportunity structures; in other words, if people have broader life aspirations that cannot be fulfilled at home, the aspiration to migrate emerges. He suggests that education in rural areas tends to increase pupils’ awareness of “alternative, consumerist, and urban lifestyles”—potentially changing their notions of the good life and introducing the aspiration to migrate (2014: 24; see also Mabogunje 1970; Rhoda 1983). As long as aspirations grow faster than the livelihood opportunities available in sending regions and countries, migration will likely continue or increase.

One limitation to the theoretical perspectives cited above is the assumption that formal education is something that is attained in origin areas, shaping the migration decision‐making process before migration occurs. This perspective neglects the well‐documented but little theorized reality that accessing education is often the first reason for rural‐urban migration in poorer countries. While primary schools are increasingly accessible to rural areas, secondary and tertiary schools may require a move to towns or cities. Complementing a broad literature on “student mobility,” Crivello (2011) refers to this movement as “migration‐for‐education” and highlights that young people often migrate at very young ages. It is after a young person (and potentially their family) moves to a more urban area for education that further movement or return is renegotiated. Thus, rather than the pursuit of work being the impetus for urbanward movement, as is often assumed, the pursuit of education drives the first migration experience of many young people.

## The Ethiopian context

### Economic, demographic, and educational transformations

Economic, demographic, and educational transformations in Ethiopia are unfolding at a rapid pace. Since the fall of the communist regime and the rise of a more market‐oriented state in the 1990s, the Ethiopian government embarked on an ambitious growth and transformation agenda that achieved economic growth rates averaging 10.8 percent per year from 2003/2004 to 2014/2015. During these years, the government shifted from an agricultural development‐led industrialization strategy, which focused primarily on increasing the productivity of rural farmers, to a strategy that gives greater emphasis to free enterprise, foreign investment, and the creation of industrial parks (Lefort 2015). Some 73 percent of the population remains engaged in agriculture, but with the gradual rise of the industry and service sectors (accounting for 7.4 and 19.9 percent of employment in 2013, respectively; UN 2017), formal and informal employment opportunities outside agriculture are increasing. Alongside these economic shifts, investment in infrastructure has increased connectivity and accessibility of urban areas. The number of people residing in or within three hours of a city of at least 50,000 rose from 15.5 percent of the population in 1984 to almost half in 2007 (Dorosh and Schmidt 2010)—and likely much higher today. Furthermore, Ethiopia is in the early stages of a demographic transition. Infant, child, and maternal mortality have fallen sharply over the past decades, while total fertility rates declined more slowly (today averaging 4.6 live births per woman). With a population now over 100 million, the median age is 18 years (UN 2017).

This sizable young generation is also the first to receive formal education on a wide scale. Although Haile Selassie focused on introducing “modern schools” to Ethiopia, particularly from the 1940s onwards, only the urban elite or lucky few had access to them. The communist Derg regime expanded formal education more widely during the 1970s and 1980s, with particular emphasis on building schools in rural areas, yet the numerical reach remained limited. Today's federal government has vigorously pursued the achievement of universal primary education since the 1990s. Reflecting ambitious policies and programs to expand the number of schools in rural areas, abolish school fees, and train new cohorts of teachers, education as a percentage of total government expenditure increased from approximately 12 percent in 1980 to 27 percent in 2013 (World Bank 2017; UNESCO 2011; World Bank/UNICEF 2009). The share of GDP spent on education, around five percent during 2003–2008, is high by international standards (Ravishankar, Kello and Tiruneh 2010). As a result of these investments, the number of primary schools increased from 9,900 in 1995 to 32,048 in 2014, and primary school net enrollment jumped from 21 percent in 1996 to 93 percent in 2014 (UN 2014). Nevertheless, with rapid expansion comes questions about declining quality related to resource constraints and teacher‐training (Akalu 2014).

Secondary and tertiary enrollment rates remain lower, at just 40 percent and 9 percent in 2014, respectively (FDRE 2016). The government has given what some describe as unbalanced attention to the growth of its tertiary education system, the sub‐sector that expanded most rapidly in recent years (Ravishankar, Kello, and Tiruneh 2010). One reason for this emphasis on higher education is to form actors for the national development process. In an effort to re‐align its higher education curricula to national development strategies, the Ethiopian government decreed in 2008 that 70 percent of all students should study science and technology subjects and asked all universities to modify their curricula accordingly (Rayner and Ashkroft 2011).

Education is widely perceived as a pathway to success through which rural and urban families alike must pass to achieve a better future (Mains 2013). As a result, young people and their families increasingly invest in education instead of agricultural or trade livelihoods (Tadele and Gella 2014). Investment in education for rural families often means supporting the rural‐urban migration of their children. Secondary and particularly tertiary schools are almost always found in urban areas, leading to “migration‐for‐education” (Crivello 2011) of rural students who pass qualifying regional and national exams. Additionally, when young women or men are unable to continue their education, or when their education fails to translate into the professional futures they desire, migration arises as an alternative pathway to achieve their aspirations (Mains 2013; Kuschminder 2017).

These social transformations foreshadow rising rates of internal and international migration in the years to come (see Zelinsky 1971; Skeldon 1990; De Haas 2010). Yet, at the same time that the Ethiopian government pursues rapid economic growth, infrastructure development, and the expansion of formal education, it fears a large influx of rural youth to urban centers, where livelihood opportunities remain limited. In urban Ethiopia, conservative evaluations of youth unemployment rates estimate 17.5 percent, with higher rates in large cities like Addis Ababa (23.0 percent) and Dire Dawa (22.7 percent) (Kibret 2014). To avoid growing urban unemployment, rural development policy proposed that at least 70 percent of rural students should be absorbed in agricultural labor (FDRE 2003). Dorosh and Schmidt (2010) suggest that land rights policies are designed to dissuade leaving rural areas through, for example, regulations prohibiting sale of land, loss of land rights for those who leave rural areas, and registration requirements for new migrants. Nevertheless, many researchers continue to highlight a strong resistance among rural youth in Ethiopia—particularly those with education—to “end up like their farmer parents” (Tadele and Gella 2012: 6; Camfield 2011; Abebe 2008).

### Migration within and from Ethiopia over the past decades

The first significant movement of international migrants from Ethiopia took place in the 1970s and 1980s after the rise of the communist Derg regime, when hundreds of thousands of political refugees sought asylum elsewhere. Emigration has continued under the current federated state yet shifted from a primarily refugee‐driven migration to a diversifying set of labor migration trajectories: within Africa, to the “Global North,” and to the Middle East (Kuschminder et al 2012). Most of those leaving for the Middle East—found to be half of those migrating in one study (Kuschminder and Siegel 2014)—are young, single, and female, responding to economic opportunities as domestic workers (Carter and Rohwerder 2016). Overall, total emigration rates from Ethiopia remain relatively low, hovering around one percent of the population since 2000 (UN 2015). Yet, this percentage becomes more significant when viewed from the lens of education levels: in 2000, 10 percent of the tertiary‐educated population had emigrated (World Bank 2011), with top destinations including the United States, South Africa, Sweden, India, and Finland (UN 2013).^3^

Internal movement is greater than international migration, although statistics remain scant due to the limited availability of nationally‐representative data. To set some context for internal migration, then, we use three rounds of Labour Force Survey data (1999, 2005, and 2013) to explore intra‐regional movement of recent migrants, aged 15 and over, who changed residence within the last five years.^4^ Ethiopia is divided into some 63 zones, which are further divided into approximately 660 rural and 100 urban districts (*woreda*).^5^ These data are nationally representative, but only capture migration across zonal borders, not movement within zones and woredas, thus underestimating the extent of internal migration and missing important processes of small‐scale urbanization happening within traditionally rural, agrarian areas.^6^ Nevertheless, these data provide an initial sketch of internal mobility patterns over the last two decades, to complement a small but growing literature on internal migration in Ethiopia (see Blunch and Laderchi 2015).

Approximately six percent of the Ethiopian population aged 15 and over had migrated across zones within the five years prior to 2013. The literacy and education levels of internal migrants generally increased since the late 1990s and were consistently higher than those of non‐migrants (Table 1). While rural‐rural migration has typically been the primary mode of movement within Ethiopia, rural‐urban and urban‐urban migration replaced migration between rural areas as the most common migration trajectory of internal migrants in the last decade (See Appendix, Table A1).

**Table 1:** Characteristics of internal migrants over time

	1999		2005		2013	
Migrant characteristics	Internal migrants	Non‐migrants	Internal migrants	Non‐migrants	Internal migrants	Non‐migrants
Age (mean)	27.22	34.70	22.56	25.63	22.72	25.43
Sex (1 = male)	0.44	0.48	0.49	0.49	0.44	0.50
Marital status (1 = married)	0.49	0.61	0.39	0.41	0.36	0.39
Literacy (1 = yes)	0.48	0.28	0.49	0.32	0.64	0.44
Years of schooling (mean)	3.40	1.45	3.18	1.48	4.74	2.35
No school attainment (1 = yes)	0.54	0.74	0.54	0.70	0.31	0.49
Primary school attainment (1 = yes)	0.29	0.21	0.31	0.26	0.48	0.44
Secondary school attainment (1 = yes)	0.13	0.04	0.10	0.03	0.18	0.06
Higher education attainment (1 = yes)	0.04	0.01	0.05	0.01	0.02	0.01

NOTES: Based on LFS data. Recent migrants are individuals who moved less than five years prior to survey data collection. Based on the population aged 15 and over. Sample sizes range between 134,000 and 199,000 per survey year.

**Figure 1:**
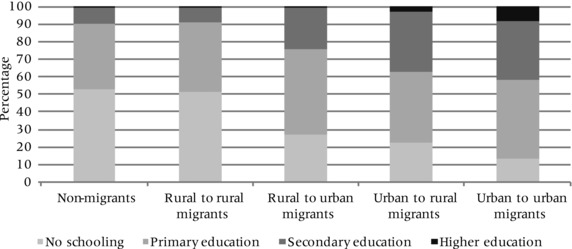
Education attainment of non‐migrants and internal migrants by migration pattern NOTES: Based on LFS 2013 data. Recent migrants are individuals who moved less than five years prior to survey data collection. Based on the population aged 15 and over. The sample includes 146,198 individuals.

In general, those moving to or leaving urban areas tend to be more highly educated than rural‐rural migrants and non‐migrants (Table 2; Figure 1). Other studies in Ethiopia have found that internal migrants tend to be more highly educated than non‐migrants (Tegegne and Penker 2016; Bezu and Holden 2014), and Blunch and Laderchi (2015) found that migrants obtain higher returns to their education than non‐migrants. These authors note that in, addition to economic opportunity, educational facilities in urban areas can be an attractive factor.

**Table 2:** Migrant characteristics by migration pattern

Migrant characteristics	Rural to rural	Rural to urban	Urban to rural	Urban to urban
Age (mean)	27.07	25.34	28.16	26.47
Sex (1 = male)	0.48	0.40	0.56	0.40
Marital status (1 = married)	0.58	0.38	0.48	0.44
Literacy (1 = yes)	0.44	0.70	0.76	0.84
Years of schooling (mean)	2.88	5.77	7.14	7.82

NOTES: Based on LFS 2013 data. Recent migrants are individuals who moved less than five years prior to survey data collection. Based on the population aged 15 and over. The sample includes 146,198 individuals.

After the search for work (59 percent men, 39 percent women), education was the most common reason given for rural‐urban migration (15 percent men, 15 percent women). While the Labour Force Survey data present migration motivations for all internal migrants over the age of 15, a study by Erulkar et al. (2006) of young people (ages 10–19) in poor areas of Addis Ababa found that around half of all boys and girls moved there primarily in pursuit of educational opportunities.

The Labour Force Survey data present an illuminating, nationally‐representative overview of internal migration patterns and the relationships between education levels and migration trajectories. To better understand how education shapes the aspiration to migrate among young people in Ethiopia, we now turn to the Young Lives survey data.

### Education and migration aspirations: Young Lives data

We use the Young Lives survey data to explore the determinants of migration aspirations among rural and urban youth in Ethiopia. In contrast to most migration aspiration surveys to date that ask only about international migration aspirations, the Young Lives data asks whether young people would like to move in the next 10 years, and if so where they would be most likely to move.^7^ By allowing for a range of responses across administrative boundaries, the survey provides a unique opportunity to consider internal and international destinations at the same time.

The Young Lives study is a longitudinal study on child and youth poverty funded by the UK Department for International Development (DfID). This project collected panel data over a fifteen‐year period in four countries—Ethiopia, India, Peru, and Vietnam—among a younger cohort (2,000 in each country) and an older cohort (1,000 in each country) of children. The first round of data collection in Ethiopia started in 2002. It is important to note that the Young Lives survey is not nationally representative but sampled to capture a wide variety of circumstances (Young Lives, 2014). In line with the overall focus of the study on childhood poverty, districts with higher food shortages were, for example, oversampled to ensure the inclusion of poor children, and urban and rural areas were purposively sampled so that children from both areas were included. Finally, the sampling of areas aimed to reflect the diversity of Ethiopia's population in terms of ethnicity and region of residence.^8^

We use the fourth round of data collected among the older cohort in Ethiopia between October 2013 and March 2014. Because of attrition, the 2013/2014 data include 909 of the original 1,000 participants. After eliminating missing observations, our analyses proceed with a final sample size of 823 individuals. Most of the participants in our sample were 18 or 19 years of age and a slight majority of the respondents (54 percent) were male. Around half of the participants (48 percent) resided in urban areas at the time of the survey, and the respondents were more or less evenly spread among the five survey regions: Addis Ababa, Amhara, Oromia, Tigray, and Southern Nations, Nationalities, and Peoples’ Region (SNNPR).

**Figure 2:**
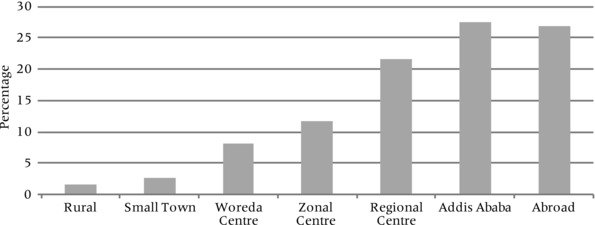
Type of most likely migration destination SOURCE: Young Lives study Ethiopia, fourth round, 2013/2014, older cohort (n=823).

Approximately two‐thirds of the youth who participated in the Ethiopia Young Lives study expressed an aspiration to migrate (see Table 3). Of these, 58 percent had a destination in mind, which in most cases was an urban location in Ethiopia. In fact, a clear gradient emerges in the migration horizons of young people surveyed, with larger urban centers attracting more young people (Figure 2). A minority of those with a migration aspiration (26 percent) expressed a wish to migrate abroad. Table 3 depicts the main motivations underlying the aspiration to migrate or to stay: the most common motivations for migration were related to employment and education, and the desire to stay was most often related to family and community considerations.

**Table 3:** Migration aspirations: descriptive statistics

Variable	Categories	Freq.	Perc.
Migration aspiration	No	262	31.83
	Yes	561	68.17
Know migration destination	No	234	41.71
	Yes	327	58.29
Main reason for wanting to migrate	Employment purposes	339	60.86
	Educational purposes	158	28.37
	Health care facilities/housing/public services	18	3.23
	Family reasons	17	3.05
	Broaden horizons/seek independence	8	1.44
	Other reasons	17	3.06
Main reason for not wanting to migrate	Have family/community here	118	45.38
	At school/studying here	43	16.54
	Have a job I like here	32	12.31
	Happy here/have a good life	26	10.00
	Have house/land/property here	10	3.85
	Other reasons	31	11.92

SOURCE: Young Lives study Ethiopia, fourth round, 2013/2014, older cohort (n = 823).

### Education and other development‐related, independent variables

To better understand the determinants of migration aspirations, we explored several variables from the Young Lives data (see Table 4). First, educational attainment was divided into four levels: no education certificate, primary education, secondary education, and those with a higher education entrance certificate.^9^ Most youth surveyed fell into the first category (37 percent), suggesting that despite rapid expansion in primary education across Ethiopia, many of the young people surveyed for the Young Lives project did not finish primary education. To distinguish current enrollment from attainment, we added an additional variable “currently in school” to capture ongoing participation in formal education (60 percent of our sample).

**Table 4:** Descriptive statistics of the main explanatory variables

Variable	Mean	Standard deviation	Min.	Max.
Age	18.50	0.55	16	20
Sex (1 = male)	0.54	0.50	0	1
Urban residence	0.46	0.50	0	1
Highest educational attainment				
No certificate	0.37	0.46	0	1
Primary school completion	0.31	0.46	0	1
Secondary school completion	0.26	0.44	0	1
Higher education entrance certificate	0.06	0.25	0	1
Currently in school	0.60	0.49	0	1
Wealth index	0.36	0.16	0.01	0.88
Relative wealth	2.89	0.90	1	5
Worked on own farm in the past 7 days	0.30	0.46	0	1
Worked on own business in the past 7 days	0.16	0.36	0	1
Worked in a paid job in the past 7 days	0.19	0.39	0	1
Moved for education since 2009	0.16	0.37	0	1
Moved for work since 2009	0.12	0.32	0	1
I can solve most problems if I invest the necessary effort	2.27	0.55	1	4
Aspired educational attainment	14.06	2.81	0	16
Aspired employment skill level	3.28	1.00	1	4

SOURCE: Young Lives study Ethiopia, fourth round, 2013/2014, older cohort (n = 823).

Second, to study the relationship between household wealth and migration aspirations, we include a wealth index and a subjective measure of wealth. The wealth index was included in the dataset and is composed of three indices measuring housing quality, consumer durables, and access to services.^10^ We also include a squared term of the wealth index to test if the relationship between wealth and migration aspirations is nonlinear. Relative wealth was measured with the question “C*ompared to other households here in your [locality], how would you describe your household?”* with answer categories from 1 (the poorest) to 7 (the richest). Because few youth had chosen the extreme categories, we recoded the variable into a five‐point scale, ranging from 1 (the poorest/among the poorest) to 5 (the richest/among the richest). The relative wealth indicator correlated slightly with the wealth index (*r = 0.14*) and both variables are therefore included simultaneously in the regression models.

Educational attainment and wealth levels are substantially correlated (*r = 0.43*). Wealth levels ranged from below average for those with no education certificate (Mean = 0.28) and rose for each subsequent education level to a mean of 0.52 for those with the higher education entrance certificate. In other words, children from wealthier families attained higher levels of education. This is not surprising as these children may be less likely to be pulled out of school to help with economic needs at home. Furthermore, secondary education is most often located in urban areas in Ethiopia; for rural families, it can require significant resources to send a child to an urban area for schooling.

Third, we study how employment is related to migration aspirations by including indicators for work: (a) on their own or family‐owned farmland, (b) for a self‐owned business, or (c) in a paid job, in the seven days prior to the survey.^11^ A key motivation for migration is to seek work opportunities (Table 3), and this indicator allows us to see whether existing engagement in different types of employment diminishes migration aspirations.

Fourth, previous migration experiences for education or employment were included with the hypothesis that previous experience with migration may increase the likelihood of aspiring to migrate again. More young people moved for education (16 percent) than for employment (12 percent) since 2009 (round 3 of the survey). Further data explorations (available upon request) revealed that those who had previously moved for education were on average more highly educated, wealthier, and more often enrolled in school than those who had not moved for education, while those who had previously moved for work had lower levels of education and were less often in school, with little difference in wealth levels.

Fifth, we include measures of educational and occupational aspirations as well as feelings of self‐efficacy, to understand how more general aspirations for the future and the confidence to achieve them relate to the aspiration to migrate. We chose three questions to reflect this interest. First, we include a measurement of education aspirations, which asked what level of education the youth aspired to attain imagining they had no constraints, ranging from 0 (no education) to 16 (a post‐graduate degree). Interestingly, the youth surveyed aspired to very high levels of education, averaging over 14 years, equivalent to a university education. Second, we include a measurement of aspired employment skill level, which asked what type of job the youth aspired to do in the future, given there were no constraints. Based on the International Standard Classification of Occupations (ISCO) scale (ILO 2012), the average here (3.28) reflects work at the skill level of professionals, managers, and technicians, among others. The last indicator relates to feelings of self‐efficacy, measured by the statement “*I can solve most problems if I invest the necessary effort,”* with answer categories ranging from 1 (strongly disagree) to 4 (strongly agree). Because few respondents opted for the “strongly disagree” category, we combined this category with the “disagree” category. Levels of self‐efficacy were in fact not low, but nor were they exceptionally high, averaging 2.27.

Although the subsequent analyses focus on those with the aspiration to migrate, it should be noted that youth who preferred to stay were slightly more likely to be male (52 percent male) and to come from a rural setting (53 percent rural). They had lower education levels (46 percent with no education certificate) than those with migration aspirations (32 percent with no education certificate) and were less often enrolled in school (50 percent versus 64 percent).

At first glance, education seems to be significantly and positively related to the aspiration to live elsewhere, with those who completed primary school or higher more often aspiring to migrate than those with no educational certificate (see Table 5). There are less significant but nuanced differences in the aspiration to migrate internally or internationally that suggest education may influence these imagined destinations differently. For example, internal migration aspirations were highest among those who completed primary school. International migration aspirations, however, were highest among those with the highest educational attainment and among those with no education certificates. Those with intermediate educational attainment less often expressed the desire to migrate abroad. Students who were currently enrolled in school were more likely to aspire to live elsewhere, but less likely to know their destination. Among those who had a clear destination in mind, however, young people who were no longer in school were more likely to aspire to migrate abroad. The following regression analyses reveal to what degree internal and international migration aspirations differ according to educational attainment and status when controlling for other variables (for further descriptive analyses of key indicators, see Table A2 in Appendix).

**Table 5:** Migration aspirations and education: descriptive statistics

Variable	Migration aspiration (n = 823)	Destination is known (n = 561)	Internal migration aspiration (n = 561)	International migration aspiration (n = 561)
Educational attainment				
No certificate	0.59	0.62	0.44	0.17
Primary school completion	0.75	0.61	0.49	0.12
Secondary school completion	0.72	0.50	0.35	0.16
Higher education entrance cert.	0.74	0.62	0.41	0.21
F‐value	6.19***	1.85	2.54*	1.19
Currently in school				
No	0.62	0.67	0.47	0.21
Yes	0.73	0.53	0.41	0.12
T‐value	–3.37***	3.35***	1.32	2.78***

^***^p<0.01, ^**^p<0.05, ^*^p<0.1.

SOURCE: Young Lives study Ethiopia, fourth round, 2013/2014, older cohort.

### Regression results

To explore how educational attainment, enrollment, and other development‐related factors relate to the aspiration to migrate, Table 6 shows the regression results for general migration aspirations (meaning the desire to migrate regardless of destination) in a step‐wise approach. Education (Model 1) and wealth indicators (Model 2) are included separately to check robustness of the results. In a third model, we include previous migration experience, the measurement of self‐efficacy, and education aspirations. Previous migration and self‐efficacy may be related to educational attainment or wealth and were therefore included separately as well. In Table 7 we apply a multinomial logit to test how youth with internal migration aspirations, international migrations aspirations, and those with migration aspirations but without a destination in mind, differ from youth who prefer to stay (the reference group).

**Table 6:** Migration aspirations (internal or international)

Variable	Model 1	Model 2	Model 3	Model 4
Age	–0.03	–0.10	–0.07	0.00
	(0.15)	(0.15)	(0.15)	(0.15)
Sex (1 = male)	0.24	0.12	0.10	0.15
	(0.16)	(0.16)	(0.16)	(0.17)
Educational attainment. (ref. = no certificate)				
Primary school completion	0.67***			0.53**
	(0.20)			(0.22)
Secondary school completion	0.57***			0.42*
	(0.21)			(0.24)
Higher education entrance cert.	0.66*			0.80*
	(0.36)			(0.43)
Currently in school	0.43**			0.49**
	(0.17)			(0.20)
Wealth index		5.34***		5.17***
		(1.91)		(1.98)
Wealth index squared		–5.95***		–6.42***
		(2.19)		(2.25)
Relative wealth		–0.00		–0.04
		(0.09)		(0.10)
Worked: farm in past 7 days		–0.09		–0.12
		(0.20)		(0.21)
Worked: business in past 7 days		0.20		0.21
		(0.23)		(0.25)
Worked: paid job in past 7 days		–0.08		0.11
		(0.21)		(0.23)
Previously moved for educ.			–0.22	–0.53**
			(0.24)	(0.26)
Previously moved for work			0.39	0.68**
			(0.26)	(0.27)
Feel able to solve most problems			0.43***	0.41***
			(0.15)	(0.16)
Aspired education			0.13***	0.08**
			(0.03)	(0.03)
Aspired employment skill level			0.21***	0.21**
			(0.08)	(0.08)
Urban residence	–0.13	–0.05	–0.08	–0.40
	(0.18)	(0.23)	(0.18)	(0.24)
Constant	0.08	1.20	–2.13	–3.85
	(2.78)	(2.72)	(2.84)	(2.94)
Number of observations	823	823	823	823
Pseudo R‐Squared	0.05	0.04	0.07	0.09

^***^p<0.01, ^**^p<0.05, ^*^p<0.1. The region of residence is controlled for.

**Table 7:** Internal and international migration aspirations (multinomial logit)

	Internal migration aspiration				International migration aspiration				Don't know location			
	(1)	(2)	(3)	(4)	(5)	(6)	(7)	(8)	(9)	(10)	(11)	(12)
Age	–0.00	–0.09	–0.06	0.03	–0.35	–0.28	–0.32	–0.35	0.12	–0.03	0.07	0.20
	(0.17)	(0.17)	(0.17)	(0.18)	(0.25)	(0.24)	(0.24)	(0.24)	(0.18)	(0.17)	(0.18)	(0.19)
Sex(1 = male)	0.39**	0.24	0.25	0.31	–0.73***	–0.70***	–0.95***	–0.89***	0.53***	0.34*	0.38*	0.48**
	(0.19)	(0.19)	(0.19)	(0.20)	(0.27)	(0.27)	(0.28)	(0.28)	(0.20)	(0.20)	(0.20)	(0.22)
Educational att. (ref. = no cert.)												
Primary school completion	0.85***			0.69***	0.37			0.08	0.59**			0.56**
	(0.24)			(0.26)	(0.35)			(0.39)	(0.25)			(0.27)
Secondary school completion	0.48*			0.30	0.45			0.03	0.68**			0.71**
	(0.26)			(0.29)	(0.37)			(0.41)	(0.27)			(0.31)
Higher education entrance cert.	0.92**			1.00**	0.85			0.48	0.30			0.71
	(0.43)			(0.51)	(0.56)			(0.63)	(0.45)			(0.52)
Currently in school	0.31			0.39	–0.42			–0.33	0.94***			0.92***
	(0.20)			(0.25)	(0.29)			(0.36)	(0.22)			(0.25)
Wealth index		4.37**		4.48*		3.65		5.42*		7.88***		6.63**
		(2.19)		(2.35)		(3.09)		(3.26)		(2.71)		(2.76)
Wealth index squared		–4.57*		–5.39**		–3.03		–5.18		–9.80***		–9.17***
		(2.56)		(2.70)		(3.64)		(3.85)		(3.20)		(3.26)
Relative wealth		–0.02		–0.03		0.18		0.21		–0.09		–0.19
		(0.11)		(0.12)		(0.16)		(0.17)		(0.11)		(0.12)
Worked: farm in past 7 days		–0.10		–0.13		–0.54		–0.62*		0.11		0.08
		(0.23)		(0.24)		(0.34)		(0.34)		(0.26)		(0.28)
Worked: business in past 7 days		0.22		0.23		–0.02		–0.26		0.24		0.40
		(0.27)		(0.29)		(0.38)		(0.40)		(0.28)		(0.30)
Worked: paid job in past 7 days		0.14		0.30		0.42		0.26		–0.60**		–0.27
		(0.24)		(0.27)		(0.32)		(0.34)		(0.26)		(0.29)
Previously moved for educ.			–0.20	–0.50*			0.42	0.20			–0.53*	–0.87***
			(0.28)	(0.30)			(0.38)	(0.40)			(0.28)	(0.32)
Previously moved for work			0.58**	0.78**			1.15***	1.13***			–0.47	–0.01
			(0.29)	(0.31)			(0.34)	(0.37)			(0.36)	(0.38)
Feel able to solve most problems			0.41**	0.39**			0.66***	0.68***			0.33*	0.28
			(0.17)	(0.18)			(0.25)	(0.25)			(0.18)	(0.19)
Aspired education			0.13***	0.09**			0.06	0.06			0.17***	0.09*
			(0.04)	(0.04)			(0.04)	(0.05)			(0.05)	(0.05)
Aspired employment skill level			0.33***	0.33***			0.02	0.09			0.16	0.11
			(0.10)	(0.10)			(0.13)	(0.14)			(0.10)	(0.10)
Urban residence	–0.06	–0.03	–0.02	–0.37	–0.13	–0.41	–0.10	–0.47	–0.22	0.07	–0.10	–0.36
	(0.21)	(0.26)	(0.21)	(0.28)	(0.34)	(0.39)	(0.31)	(0.39)	(0.23)	(0.28)	(0.22)	(0.30)
Constant	–1.16	0.35	–3.45	–5.50	5.90	3.59	2.80	2.05	–4.98	–2.11	–6.56*	–8.91**
	(3.23)	(3.15)	(3.32)	(3.43)	(4.70)	(.4.65)	(4.57)	(4.63)	(3.40)	(3.30)	(3.45)	(3.67)
Number of observations	823	823	823	823	823	823	823	823	823	823	823	823
Pseudo R‐Squared	0.08	0.07	0.09	0.12	0.08	0.07	0.09	0.12	0.08	0.07	0.09	0.12

***p<0.01, **p<0.05, *p<0.1.

NOTE. The reference group includes those without migration aspirations. The region of residence is controlled for.

The findings in Table 6 on general migration aspirations suggest that the desire to move elsewhere is significantly influenced by educational attainment, such that those who completed primary school or higher are more likely to aspire to migrate than those with no education certificate. Furthermore, those enrolled in school at the time of the survey were more likely to aspire to move elsewhere. Household wealth levels (and its squared term) also have a strong effect on the desire to migrate, with youth who are “in the middle” of the wealth distribution showing the greatest aspirations to leave. This bell‐shaped relationship between wealth and migration aspirations challenges common assumptions in public and popular discourse that it is the poorest who are “pushed” to migrate, but neither is it the wealthier who likely have greater capabilities to leave. Relatedly, youth engaged in recent employment—in agriculture, business, or paid work—were no different than unemployed youth in their migration aspirations, challenging an associated development assumption that generating employment in local areas should reduce the desire to leave.

Youth who had greater feelings of self‐efficacy, as well as those with higher education aspirations and an orientation toward more professional forms of work, were more likely to aspire to move elsewhere. These findings suggest that more general aspirations for the future, and the confidence to achieve them, are forces driving the aspiration to migrate. Previous experience with migration varied in its influence on the aspiration to do so again. If youth had already migrated for education, they were less likely to aspire to leave again, perhaps because they were already in an urban area where they could envision further education and livelihood opportunities. However, youth who had previously moved for work were more likely to aspire to leave again.

**Figure 3:**
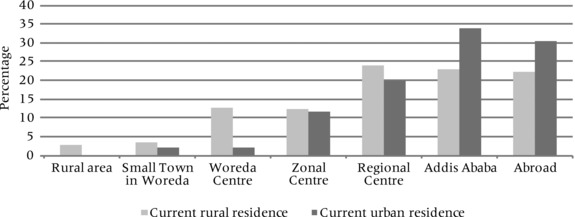
Type of most likely migration destination, by current residence SOURCE: Young Lives study Ethiopia, fourth round, 2013/2014, older cohort (n=823)

Finally, general migration aspirations do not appear to differ significantly across age groups or sex, though youth currently living in rural areas are more likely to aspire to leave their location than their urban counterparts. Mapping aspired destinations by current location suggests that youth in urban areas tended to imagine moving to larger urban destinations, particularly the capital city Addis Ababa, while youth in rural areas often looked to local or regional urban centers (see Figure 3).

These findings hold for the desire to leave at a general level, but as Table 7 shows, some of these findings shift when we distinguish between aspired destinations. The trends regarding internal migration aspirations largely confirm those found in Table 6. Youth who completed primary school are more likely to want to move within Ethiopia than those without primary levels. In addition, youth with greater feelings of self‐efficacy, higher education aspirations and those who aspire toward work at higher skill levels are more likely to aspire to move internally. The bell‐shaped relationship between migration aspirations and wealth also holds for internal migration aspirations, confirming that those youth in the middle of the wealth distribution show the greatest aspirations to leave.

International migration aspirations show more varied trends. It should be noted that only 16 percent of the sample aspired to migrate abroad, which is an important finding when much of the public and policy discourse on migration assumes that all those in poor countries want to move to rich ones. Interestingly, education (at the primary and secondary level) is not a significant predicator of an international migration aspiration, nor were aspired education or employment levels, current employment or enrollment, or rural/urban residence. Rather, the strongest predicators of the aspiration to migrate abroad were sex, previous experience moving for work, and feelings of self‐efficacy. While sex did not predict the general desire to migrate (Table 6), men appear to be more likely to aspire to move within Ethiopia or simply “elsewhere” without a clear destination in mind, whereas women are more likely to want to migrate abroad. Of all youth in the sample, 22 percent of women aspired to migrate internationally, whereas only 10 percent of men aspired to do so. This finding likely reflects current migration trends, where opportunities to migrate internationally are gendered; an increasingly common international trajectory, for example, is women migrating as domestic workers to the Middle East or to Sudan (Kuschminder et al 2012).

The consistent influence of feelings of self‐efficacy is notable. The belief that one can overcome obstacles if one invests the necessary effort is a strong predictor of the aspiration to migrate internally and internationally. Migration aspirations, then, likely reflect greater confidence in one's self and one's future and represent a key strategy that young people use to navigate their way to a better future. Separate analyses show that feelings of self‐efficacy significantly increase with each education level (results are available upon request). The consistent influence of this variable points to the fact that beyond socioeconomic circumstances, individual characteristics and beliefs are important factors influencing the aspiration to migrate.

## Discussion

Participation in formal education affects where young people in Ethiopia see their futures, and more often than not it is elsewhere—an urban center, and for a minority, abroad. Over two‐thirds of young people surveyed in the Young Lives study desired to leave their current location, most often with the hope of achieving professional work or further educational opportunities within Ethiopia. Even though our aspirations data are not nationally representative, the observation that young people increasingly eschew rural, agrarian livelihoods is supported by our findings.

The nationally‐representative Labour Force Survey data confirms that, even across longer distances within Ethiopia and taking older adults into account, education is one notable reason for migration, and those with higher levels of education migrate more. The Young Lives data suggest that this trend is not only a reflection of differing capabilities or household wealth. Participation in formal education is one notable force shaping the aspiration to live elsewhere. Taking migration aspirations as a whole, clear patterns emerge; most relevant to this study, higher educational attainment—even simply the completion of primary school—increases the likelihood of aspiring to move elsewhere. Wealth, economic and occupational aspirations, and feelings of self‐efficacy also shape the desire to leave, yet their influence varies depending on the aspired migration trajectory: whether it was to a destination within Ethiopia or abroad.

For those looking toward destinations within Ethiopia, youth who had completed primary education were more likely to aspire to migrate compared to those with less or no education. Furthermore, youth who aspired to higher levels of education and more professional forms of work—and believed that they could overcome obstacles that arose in their path toward achieving those—were also more likely to aspire to leave. These findings challenge assumptions about the motivations behind rural‐urban and international migration in poor countries. While public and policy discourse paints migration as driven by poverty, desperation, or other “push factors” to be remedied through development aid (see Clemens and Postel 2017), there is a more nuanced relationship between wealth, education, future hopes, and the aspiration to migrate. Concerning wealth, for example, the poorest were more likely to prefer to stay where they were than those with medial levels of wealth. Likewise, those with little or no education were also more likely to prefer to stay. These findings lend support to the hypothesis proposed by De Haas (2007) that higher levels of development—here referring to higher levels of income and education—may increase the aspiration to migrate. Nevertheless, it is intriguing that those with the highest wealth levels (and likely the greatest capability to leave) are less likely to aspire to migrate internally than those “in the middle.” Some reasons may be that those with greater wealth are more content, have more location‐specific economic ties or advantages (Fischer et al 1998), and/or have the capability to achieve their broader life aspirations where they are (Schewel 2015).

The aspiration to migrate abroad was, surprisingly, less clearly influenced by education, wealth, or occupational and education aspirations, yet sex had a powerful influence. This may be explained in part by the diversifying set of international migration trajectories pursued by young people in Ethiopia today. The demographic characteristics of migrants seeking further education or high‐skilled work in destinations across Europe or North America is likely very different from those looking toward domestic work in the Middle East or irregular migration to South Africa, for example (Kuschminder et al 2012). Our findings would benefit from the ability to distinguish between the desired international destination, to understand the varying influence of education and wealth on these different types of migration aspirations.

Our findings both confirm and extend the explanations migration theories offer about the relationship between education and migration, particularly the general prediction that higher levels of education should increase the likelihood of migration, at least internally. Classical migration theories generally explain that, through the lens of migration decision‐making, education boosts the expected economic returns and thus likelihood of leaving; and from the angle of labor market structures, the skilled work that higher levels of education promise are generally dispersed across urban areas. However, our finding that migration aspirations increase after completing the *primary* level is perhaps surprising, because students remain low‐skilled. This may be explained by the fact that Ethiopia has relatively high returns to schooling. A World Bank report found that each additional year of schooling leads to an 18.5 percent increase in labor market earnings in Ethiopia, with the highest returns for completing primary education (32.7 percent), followed by tertiary (17 percent) and then secondary (16.2 percent) (Montenegro and Patrinos 2014). It is also likely that formal schooling contributes to a more profound shift in aspirations that extends beyond rational economic calculations. For example, Maurus’ (2016) research among agro‐pastoral societies in southern Ethiopia suggests that through “the influence of schooling, young people's concept of time shifts from a cyclical one, concentrated on the reproduction of the social world, towards a linear one, focused on personal and ‘national’ development” (2016: abstract). Tadele and Gella's (2014) work in two rural sites of Amhara and SNNPR showed that boys and girls came “to see how backward and traditional the lives of their parents are, as a result of their education” (2014: 37). Such explorations beyond the economic frame—to the forces that shape notions of the good life and good work more broadly—can further illumine our understanding of the impact of education on changing aspirations and migration trajectories.

Finally, a note on the still somewhat nebulous links between aspirations and behavior. In contrast to the development literature on “low aspirations” and “aspirations failure” in poor countries and in Ethiopia (see Bernard et al 2011; 2014), young people in our sample expressed high future ambitions—reflecting the spirit of optimism we tend to associate with the period of youth (Tafere 2014). Most young people surveyed believed they could solve the problems they faced if they put in the necessary effort, showed remarkably high educational aspirations, often to university degrees, and the desire for professional work thereafter. Nevertheless, despite significant progress in the expansion of Ethiopia's public education system, over one‐third of youth surveyed had not completed primary levels. This disparity highlights the tension inherent in survey questions that ask about aspirations for the future and the objective constraints people face to realize those aspirations.

Concerning migration, there is inevitably a discrepancy between those who express an aspiration to migrate and those who actually do. The degree to which the aspiration to migrate translates into actual migration depends on context‐specific obstacles and opportunities, which vary across social groups (Carling and Schewel 2018). Many of those who aspire to migrate may not realize their aspirations—perhaps because they lack the capability to move or develop other conflicting aspirations, desires, and goals. Individual aspirations are also mediated by family contexts; the decision to leave or stay may be taken at a household rather than individual level (Stark 1991). Further, many who do not aspire to migrate may find themselves on the move in response to an unexpected opportunity or change in circumstance (Lu 1999; De Groot et al 2011). Two‐step approaches to migration research, which distinguish the evaluation of migration and the realization of mobility or immobility at a given moment (Carling and Schewel 2018), hold promise for disentangling the links between aspirations and actual migration behavior. Docquier, Peri, and Ruyssen (2014), to give one example, in a cross‐country analysis of the determinants of “potential” and “actual” international migration, find that tertiary education levels are one strong predictor of whether “potential” migration becomes realized. As research on aspirations develops in the migration and development fields, the links between aspirations and behavior is an important area for further conceptual and empirical consideration.

Nevertheless, we argue that aspirations remain relevant subjects of inquiry in their own right; capturing the aspirational horizons of these youth illumines the hopes and goals that guide their life choices and signals where young people envision their futures. It is one lens into broader shifts in a population's social imaginary (Taylor 2002; Steger and James 2013). And practically, if widespread migration aspirations remain unfulfilled and lead to “involuntary immobility,” this can have negative consequences for the development of individuals and communities (see Carling 2002; Carling and Schewel 2018).

## Conclusion

This paper asks where young people in Ethiopia envision their futures and how the experience of formal education shapes the desire to live elsewhere. Unique in its ability to reveal gradients of internal and international migration aspirations, the Young Lives data shows that most youth aspire to an urban future within Ethiopia, and that these aspirations are shaped at the level of primary and secondary schooling. For a country where some 80 percent of the population still live in rural areas, the overwhelmingly urban aspirations of young people surveyed in this study are striking.

Many countries worldwide are pursuing the expansion of formal education at unprecedented levels. This paper proposes that this admirable development accomplishment has mobility consequences. Formal education is one force, among others, shaping the migration trajectories of young people in two important ways: (1) structurally, because secondary and higher education is often only found in urban centers and thus the pursuit of higher education requires migration for rural youth; and (2) aspirationally, because the experience of formal education and the professional opportunities it promises shape young people's notions of the good life, good work, and expectations about where these might be achieved. While the aspiration to migrate requires certain capabilities to be realized (see Carling 2002; De Haas 2014; Carling and Schewel 2018), these aspirational shifts signal an important transformation in the social imagination of Ethiopia's young people.

Our findings have important implications for migration research and development policy. First, our findings challenge assumptions behind development strategies to “address the root causes of migration” (e.g. European Council 2017; ILO 2017; see also De Haas 2007; Clemens and Postel 2017). Development agendas that aim to keep people “on the farm” (Rhoda 1983; Bakewell 2008) and at the same time provide higher levels of education are in tension. This study shows that widening access to formal education, even at the primary level, tends to increase the aspiration to leave. Relatedly, we find that the migration aspirations of youth with some form of recent employment were no different than their unemployed peers, calling into question the idea that generating employment in local areas should reduce the desire to leave (see, for example, ILO 2017). We also find that the poorest are actually more likely to prefer to stay where they are, challenging the (often implicit) notion that poverty levels and the aspiration to migrate are somehow linearly correlated. To better understand the nuanced relationship between migration and development, research should explore the determinants of the gap between aspirations and local livelihood options. We argue that the rapid expansion of mass formal education in the modern period is one significant but overlooked factor shaping this gap and thus migration trends, trajectories, and imaginaries the world over.

Second, our analyses show that the general aspiration to migrate and more specific internal or international migration aspirations have different determinants. For example, while sex did not predict the general aspiration to migrate in our first analysis, it did significantly predict migration aspirations when differentiating internal versus international destinations. Women were more likely to aspire to migrate abroad, while men were more likely to want to move, without knowing exactly where. As King and Skeldon (2010) argue, to study only internal or international migration lends a partial analysis of the drivers of migration. Empirical studies that examine the determinants of internal and international migration together can enrich our understanding of the dynamics of each.

Finally, the relationship between formal education and migration aspirations we find in this study is not necessarily fixed. Unaddressed here, but certainly relevant for further research, is how different forms of schooling influence conclusions about the relationship between education and migration. Different kinds of education—for example, rural education initiatives (see Kwauk and Robinson 2016); vocational, trade, or technical diplomas; certifications and continuing education—likely shape migration aspirations and trajectories in different ways. Also relevant is how education shapes migration aspirations and behavior over the life‐course, or even across generations. Our conclusions suggest that expanding opportunities for secondary schooling to rural areas might decrease the immediate need to migrate for education; however, the gradual accumulation of human capital among rural residents may also increase the likelihood of their out‐migration in the long‐term (see Massey et al 2010). How different forms of education influence aspirations and capabilities to migrate over time is an important question for future studies to consider.

## Notes

1 Gross enrollment ratios (GER) refer to total enrollment in primary education, regardless of age, expressed as a percentage of the population of official primary education age. GER often exceeds 100 percent due to the inclusion of over‐aged and under‐aged students because of early or late school entrance and grade repetition.

2 Taylor (1987), for example, found that returns to schooling in urban areas of Mexico are higher than in the United States, where the dominant labor demand is for low‐skilled or unskilled labor. Dustmann and Glitz (2011) argue that immigrants face a lower return to schooling in the US than natives do; their wages rise more slowly with education attainment as compared to natives.

3 Kuschminder et al (2012) found that migrants in the Global North had, on average, 13 years of education versus eight to nine years among those who had migrated within Africa or to the Gulf States. Furthermore, migration for education was a motivation of 15 percent of emigrants in the Global North versus less than one percent of those abroad in Africa or the Gulf States.

4 Source of data: Labour Force Survey 1999, 2005 and 2013–March 2014, provided by the Central Statistical Authority of Ethiopia.

5 Urban centers are defined as localities with 2,000 or more inhabitants. For the purpose of the LFS survey, urban centers include (a) all administrative capitals, (b) municipal towns not included in (a), (c) localities with a population of 1,000 or more persons not included in (a) or (b) having a population of 1,000 or more and whose inhabitants are primarily engaged in non‐agricultural activities (FDRE, 2014).

6 Another important limitation of the data was that the survey questions only referred to the latest migration episode of the individual. This makes it impossible to derive information on recurrent or multiple migration episodes and the exact directions of internal migrations in Ethiopia. From the survey data, we only know where people were born and where they moved to in their most recent migration, but we do not have information on potential migration episodes that took place in between these two moments.

7 Unlike earlier survey rounds, the fourth round of the Young Lives survey collected additional information on migration aspirations. The survey asked, *“Would you like to move from your current location to a different place at some point within the next 10 years?”* and if yes, respondents were asked “*Do you know where you would like to move?”* (yes/no) and “*If you were to move, where would you be most likely to move to?*” The language of the initial question suggests the migration aspiration captured here reflects a general desire or wish to move in the future but does not specify whether this desire has translated into an intention or plan to leave. Thus, it does not say much in itself about the likelihood or capability of the individual to migrate. Nevertheless, the follow up question asks where the respondent would be “most likely” to move, introducing some degree of reflection about possible futures.

8 Within each of a total of 20 purposively sampled areas across the country, the researchers randomly selected 50 households that had a child between the age of seven and one half and eight and one half in 2002 (Outes‐Leon and Sanchez 2008). According to the Young Lives sampling report (Young Lives, 2014), the response rate for the first round of data collection was 100 percent. The attrition rate between the first and fourth round of the Young Lives survey was 8.4 percent for the older cohort (Young Lives, 2014). Attrition was due to migration of the household (n = 64), refusals to participate in later rounds (n = 12), death of participants (n = 8) and because households were untraceable (n = 7).

9 Those with no education certificate either had no formal schooling or some years of primary school before dropping out. Primary education in Ethiopia is divided into two cycles, from grades 1–4 and 5–8. Thus, the second category, primary education completion, pertains to those who finished grade 8, when a regional exam is usually offered to pupils to determine whether they may proceed to higher levels. Secondary education ends at grade 12, after which a national exam is administered to obtain a higher education entrance certificate. Only those youth who pass have the possibility of continuing to university (5 percent of our sample). Unfortunately, the dataset used did not collect data on vocational or technical diplomas, an education option increasingly pursued by youth who fail out of the formal education pathway described above.

10 See Outes‐Leon and Sanchez (2008) for more information on the wealth index construction.

11 To avoid a seasonal bias in the method collection, we confirmed that 78 percent of those who had worked on the farm in the past seven days had also worked on the farm in the past 12 months.

## Appendix

**Table A1:** Migration patterns by sex: 1999, 2005, and 2013

	1999		2005		2013	
Migration patterns (%) of recent migrants	M	F	M	F	M	F
Rural to rural	31.19	39.14	35.70	41.97	24.08	22.32
Rural to urban	21.79	21.58	27.60	23.91	29.42	37.26
Urban to rural	21.65	13.08	16.02	11.09	23.15	14.79
Urban to urban	24.37	25.56	17.78	20.43	23.35	25.63
Total migrations	100.00	100.00	100.00	100.00	100.00	100.00

NOTES: Based on LFS data. Recent migrants are individuals who moved less than five years prior to survey data collection. Based on the population aged 15 and over. Sample sizes range between 134,000 and 199,000 per survey year.

**Table A2:** Migration aspirations, wealth and employment: descriptive statistics

	Migration aspiration (n = 823)	Destination is known (n = 561)	Internal migration aspiration (n = 561)	International migration aspiration (n = 561)
**Sex**				
Female	0.67	0.62	0.40	0.22
Male	0.69	0.55	0.45	0.10
T‐value	–0.85	1.74^*^	–1.15	4.03^***^
**Urban residence**				
Rural	0.69	0.59	0.46	0.13
Urban	0.68	0.57	0.40	0.18
T‐value	0.30	0.48	1.52	–1.43
**Wealth index**				
Very low wealth	0.63	0.68	0.53	0.15
Low wealth	0.72	0.52	0.38	0.14
High wealth	0.73	0.52	0.40	0.12
Very high wealth	0.66	0.64	0.43	0.21
F‐value	2.27^*^	3.90^***^	2.53^*^	1.48
**Relative wealth**				
Poorest/among the poorest	0.71	0.55	0.45	0.10
Poorer than most	0.67	0.54	0.36	0.18
About average	0.68	0.57	0.43	0.14
Richer than most	0.75	0.68	0.51	0.16
Richest/among the richest	0.58	0.68	0.36	0.32
F‐value	1.05	1.11	1.05	1.91
**Worked on own farm in past 7 days**				
No	0.70	0.59	0.42	0.17
Yes	0.65	0.58	0.47	0.11
T‐value	1.36	0.21	–1.11	1.82^*^
**Worked on own business in past 7 days**				
No	0.67	0.59	0.43	0.16
Yes	0.72	0.57	0.46	0.12
T‐value	–0.98	0.38	–0.30	0.93
**Worked in a paid job in past 7 days**				
No	0.68	0.56	0.42	0.14
Yes	0.68	0.70	0.50	0.20
T‐value	0.05	–2.57^**^	–1.55	–1.39
**Moved for education since 2009**				
No	0.68	0.57	0.43	0.14
Yes	0.67	0.65	0.46	0.19
T‐value	0.34	–1.44	–0.61	–1.13
**Moved for work since 2009**				
No	0.68	0.55	0.42	0.13
Yes	0.72	0.79	0.52	0.27
T‐value	–0.79	–3.98^***^	–1.65	–3.15^***^
**I can solve most problems if I invest the necessary effort**				
Strongly disagree/disagree	0.48	0.59	0.36	0.23
Agree	0.67	0.57	0.44	0.13
Strongly agree	0.74	0.60	0.42	0.18
F‐value	7.03^***^	0.17	0.32	1.54
**Aspired education**				
Low	0.57	0.63	0.43	0.20
Medium	0.71	0.60	0.45	0.15
High	0.75	0.51	0.39	0.12
F‐value	9.56^***^	2.30	0.62	1.77
**Aspired employment skill level**				
1	0.56	0.45	0.27	0.18
2	0.60	0.57	0.39	0.18
3	0.76	0.69	0.51	0.17
4	0.72	0.59	0.45	0.14
F‐value	4.53^***^	1.06	1.56	0.49

^***^p<0.01, ^**^p<0.05, ^*^p<0.1.
